# A regulatory role for the redox status of the pepino mosaic virus coat protein

**DOI:** 10.1371/journal.ppat.1011732

**Published:** 2023-10-18

**Authors:** Eduardo Méndez-López, Miguel A. Aranda

**Affiliations:** Centro de Edafología y Biología Aplicada del Segura (CEBAS)-CSIC, Department of Stress Biology and Plant Pathology, Campus Universitario de Espinardo, Murcia, Spain; Agriculture and Agri-Food Canada, CANADA

## Abstract

Cysteine oxidations play important regulatory roles during animal virus infections. Despite the importance of redox modifications during plant infections, no plant virus protein has yet been shown to be regulated by cysteine oxidation. The potexvirus pepino mosaic virus (PepMV) is pandemic in tomato crops. Previously we modeled the structure of the PepMV particle and coat protein (CP) by cryo-electron microscopy and identified critical residues of the CP RNA-binding pocket that interact with the viral RNA during particle formation and viral cell-to-cell movement. The PepMV CP has a single cysteine residue (Cys127) central to its RNA binding pocket, which is highly conserved. Here we show that the Cys127Ser replacement diminishes PepMV fitness, and that PepMV CP^WT^ is oxidized *in vivo* while CP^C127S^ is not. We also show that Cys127 gets spontaneously glutathionylated *in vitro*, and that S-glutathionylation blocks *in vitro* the formation of virion-like particles (VLPs). VLPs longer than 200 nm could be formed after *in planta* CP^C127S^ overexpression, while very short and dispersed VLPs were observed after CP^WT^ overexpression. Our results strongly suggest that the CP redox status regulates CP functions *via* cysteine oxidation.

## Author summary

Viral genomes encode a limited number of proteins most of which are multifunctional. Post-translational modifications often provide the switch from one function to another, and these include phosphorylation, glycosylation, lipidation or ubiquitination, as has been described for viruses in general, and plant viruses in particular. No plant virus protein has been shown to be modified by cysteine oxidation, a redox-dependent modification, in spite of the well-known and well-described importance of cellular redox alterations during plant infections. Here we show that the only cysteine residue of the pepino mosaic virus (PepMV) coat protein (CP), which lies within the CP RNA binding pocket and is conserved among members of the virus family, can be reversibly oxidized, and both, the reduced and oxidized forms coexist *in planta*. Cysteine 127 to serine replacement results in a virus variant which has *in planta* reduced fitness, and S-glutathionylation of the cysteine residue blocks the formation of viral particles *in vitro*, while the C127S substitution makes the protein insensitive to S-glutathionylation and its *in planta* overexpression results in abundant formation of virus-like particles. Our results are compatible with a model in which the redox status of the PepMV CP regulates protein functions, including particle formation.

## Introduction

Due to their generally small sizes, viral genomes encode a reduced repertoire of proteins that are necessarily multifunctional. The switch from one function to another requires tight regulation, and this can be achieved through post-translational modifications, as has been shown for animal viruses in a number of cases [1–3]. For plant viruses, the number of examples is much narrower, and include protein modifications such as phosphorylation, glycosylation, lipidation or ubiquitination [4–7]. Cysteine oxidations, which are redox-dependent post-translational modifications, have been shown to play regulatory roles for animal virus and host proteins during infections [8–14]. No plant virus protein has been shown to be modified by cysteine oxidation, in spite of the well-known and well-described importance of redox alterations during plant infections [15,16].

A significant proportion of plant viruses have flexible filamentous particles. The flexible filamentous viruses have positive-sense, single-stranded RNA (ssRNA(+)) genomes which are encapsidated in non-enveloped particles [17,18] of about 10–15 nm in diameter and several hundred nm long, depending on the length of the genome. Their particles are built by oligomerization of viral coat protein (CP) subunits that are arranged in a helical manner to protect the viral genome. Flexible filamentous viruses infect plants and are found in 4 families: *Alphaflexiviridae* (65 species of which 48 belong to the genus *Potexvirus*), *Betaflexiviridae* (128 species), *Closteroviridae* (57 species) and *Potyviridae* (237 species of which 195 belong to the genus *Potyvirus*) [19]. The particle structures of 4 potexviruses [20–23] and 3 potyviruses [24–26] have been determined by cryo-EM, and they show almost identical architectures, despite sharing a low CP sequence identity [24]. The CPs of flexible filamentous viruses have three major regions: an N-terminal flexible arm and a C-terminal extension responsible for the lateral and axial CP-CP interactions within the virion, respectively; and an alpha helix-rich core region with a conserved RNA binding pocket. Their structural homology suggests that CPs from these viruses may have a common evolutionary origin [19,27], and point towards the RNA binding domain as a target for the design of broad-spectrum antiviral strategies. The core region, the position of the RNA binding domain, and the side-by-side CP oligomerization mediated by the N-terminal flexible arm, are also structurally conserved in nucleoproteins from representative members of the families *Phenuiviridae* (formerly *Bunyaviridae*) and *Orthomyxoviridae*, which are enveloped viruses with segmented negative-sense ssRNA genomes that cause human diseases [1,19,24].

Members of the *Potexvirus* and *Potyvirus* genera are of particular significance to agriculture as pathogens [28]. Pepino mosaic virus (PepMV) is a potexvirus that causes a disease that affects tomato crops worldwide, resulting in important economic losses. Since the first description of PepMV infection in tomato plants in 1999 [29,30], a significant amount of information on its biology has been generated, including the modelling of its particle and CP structures by cryo-EM, as well as its potential use as a nanobiotechnological tool [1,31]. The PepMV genome is a 6.4 kb ssRNA(+) molecule with a 5’-cap, a 3’ poly-A tail, and 5 open reading frames (ORFs) flanked by 5’ and 3’ untranslated regions (UTRs). The ORFs encode an RNA-dependent RNA polymerase (RdRp), the triple gene block (TGB) proteins 1, 2, and 3, and the CP. The CP, apart from its structural role, is essential for the viral cell-to-cell movement and acts as an RNA silencing suppressor, but it is not needed for viral replication [32,33]. Here we show that the PepMV CP residue Cys127 is located in the CP RNA-binding pocket and is highly conserved in the CPs of flexuous filamentous viruses; consistent with this, a Cys127Ser replacement results in reduced viral fitness. Our results also show that Cys127 is *in vivo* oxidized, and glutathionylation of this residue blocks the *in vitro* formation of virus-like particles (VLPs). Coherently, *in planta* overexpression of a Cys127Ser CP mutant efficiently produced VLPs, while overexpression of the wild type CP produced very short and isolated VLPs. These findings strongly suggest that the redox status of the only CP cysteine in the RNA-binding pocket of the protein regulates CP functions; this is the first description of a plant virus protein regulated by an oxidative post-translational modification.

## Results and discussion

**Fig 1:**
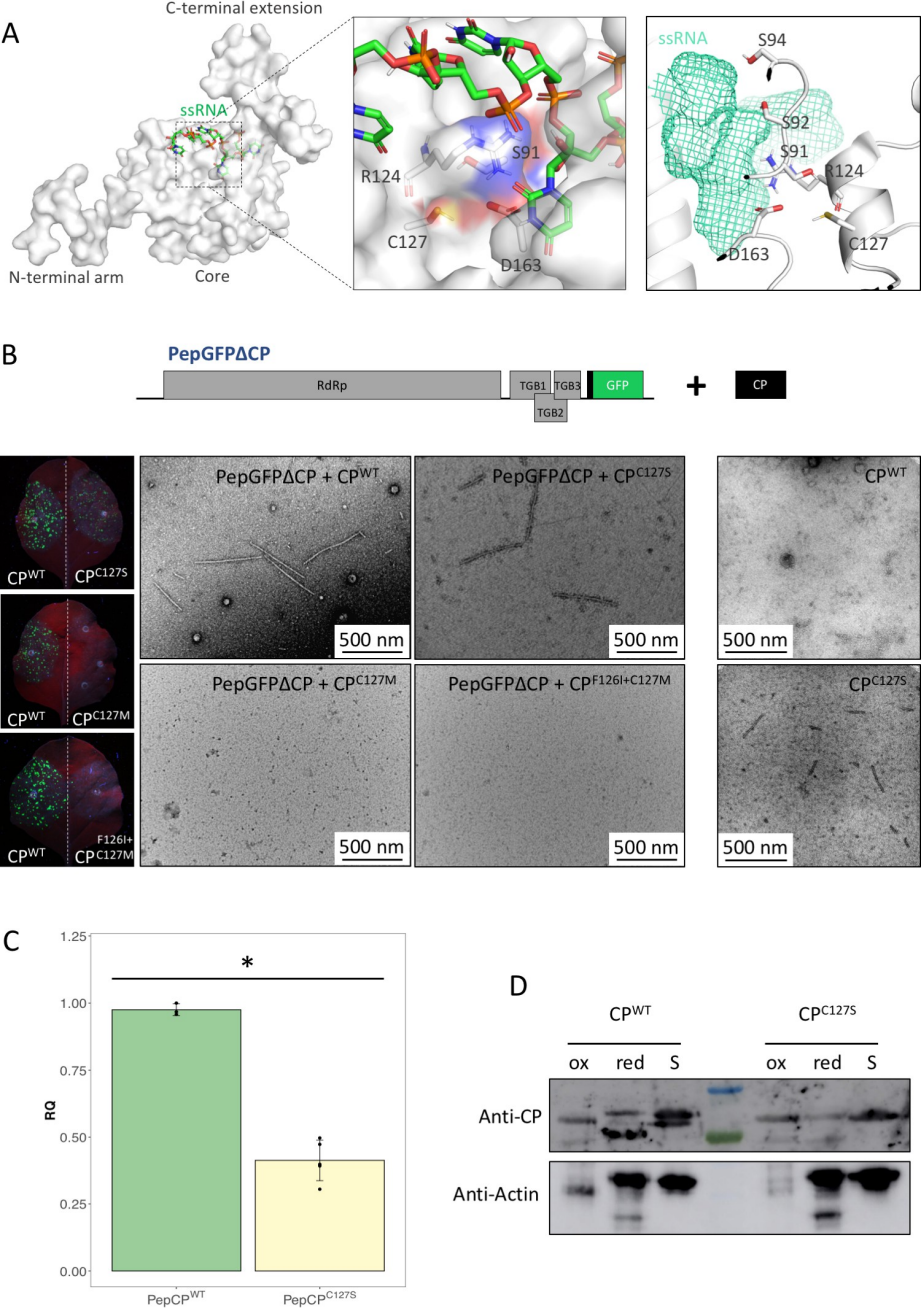
The conserved Cys127 residue of the PepMV coat protein (CP) RNA binding pocket is essential for efficient infection and is oxidized *in vivo*. (**A**) Atomic model of the PepMV CP binding to a single stranded RNA (ssRNA) molecule (pdb code 5fn1) [1] and a close-up view of the RNA-binding pocket within the virion. The CP surface is shown in semi-transparent mode and the three CP domains are labeled. A close-up view of the framed region is shown. The ssRNA and the side chain of residues Ser91, Arg124 (also the main chain), Cys127 and Asp163 are represented by sticks. Atoms are differently colored on sticks and the CP surface. On the right side of the panel, a different orientation of the RNA binding pocket is shown. The ssRNA is represented in mesh mode and the CP in cartoon mode. Residues Ser92, Ser94, Arg124 and Asp163 forming the clamp binding the ssRNA are represented by sticks, as well as residues Ser91 and Cys127. **(B)** *Trans*-complementation assays between PepGFPΔCP and three CP mutants. PepGFPΔCP [32] is a PepMV-based vector expressing the green fluorescent protein (GFP) instead of the CP. A schematic representation of the genomic organization of PepGFPΔCP and its co-expression with the CP is shown. RdRp: RNA dependent RNA polymerase; TGB1, 2 and 3: Triple gene block 1, 2 and 3 protein-encoding genes. The left side of the panel shows images of *Nicotiana benthamiana* leaves agroinfiltrared with PepGFPΔCP + CP wild type (CP^WT^) on one half of the leaf, and PepGFPΔCP + CP mutant (CP^C127S^, CP^C127M^ or CP^F126I+C127M^) on the other. Leaves were imaged 4 days post agroinoculation under UV light to detect the expression of GFP. Formation of fluorescent foci indicating the restoration of the viral cell-to-cell movement were observed when PepGFPΔCP was complemented with both CP^WT^ and CP^C127S^ mutant. Crude sap from agroinfiltrated *N*. *benthamiana* leaves from the *trans*-complementation assay were subjected to immunosorbent electron microscopy (ISEM). The micrographs show the formation of viral particles in the complementation assay with both CP^WT^ and CP^C127S^ mutants. CP^WT^ or CP^C127S^ were also expressed alone in *N*. *benthamiana* leaves, and short virus-like particles were immunocaptured by ISEM from the extract from leaves expressing CP^C127S^, suggesting that the CP^C127S^ mutant has some ability to self-assemble in the absence of the vRNA. A scale bar is drawn in each micrograph, and represents 500 nm. (C) A full-length PepMV-based clone bearing a mutation resulting in the replacement Cys127Ser in the CP (PepCP^C127S^) was generated. Bar plot showing the relative accumulation of PepCP^WT^ and PepCP^C127S^ RNAs in *N*. *benthamiana* plants 10 days post inoculation as measured by RT-qPCR. Error bars represent the standard deviation. An asterisk indicates statistically significant differences between groups, as computed by a one-way ANOVA (*F*_1,6_ = 148.61, *P* = 0.00). (D) Western blot of protein extracts from *N*. *benthamiana* leaves expressing CP^WT^ or CP^C127S^ incubated with methyl-polyethylene glycol-maleimide (MM(PEG)_24_) as described by Pant et al. (2021). Reduced cysteines are alkylated with MM(PEG)_24_ and one conjugation increases the protein mass by 1.24 kDa. Pre-oxidized (ox) and pre-reduced (red) protein extracts are included as controls. An increase in the CP^WT^ molecular weight in the reduced protein extract control is observed due to MM(PEG)_24_ conjugation. In the sample (S), a double band can be observed, indicating that residue Cys127 is oxidized *in vivo*. For CP^C127S^, no molecular weight shift was observed under any condition, as it does not contain any cysteine residue. The membrane was stripped and re-probed with an anti-Actin antibody as the loading control.

The PepMV CP RNA binding pocket is conserved among all flexible filamentous viruses, and contains a pattern consisting of a serine, an arginine and an aspartic acid that interact with the vRNA within the virion [24]; these are Ser92, Arg124, and Asp163, respectively, in the PepMV CP sequence. Ser92 interacts with the phosphate backbone of the vRNA, and Arg124 and Asp163 are at the bottom of the RNA binding pocket and interact with the base of a vRNA ribonucleotide [1]. The PepMV CP RNA binding pocket also contains a cysteine residue (Cys127) that may establish contacts with Arg124 and Asp163, and a hydrogen bond with Ser91 (S1 Table) that may stabilize the loop containing Ser92 and Ser94 within the virion structure [1] (Fig 1A). A loose CP subunit model has been proposed, in which Cys127 was predicted to only interact with Asp163, suggesting that Cys127 may not participate in stabilizing the RNA-binding domain, with its pKa dropping from 15.76 to 11.58 in the loose CP subunit model (S1 Table). Cys127 is conserved within the *Alpha*- and *Betaflexiviridae* families, except for 4 and 7 members, respectively (S1 Fig). Members of the family *Potyviridae* have a methionine residue in this position (S1 Fig). Methionine, like cysteine, can form bonds with aromatic residues, and is a sulfur-containing residue that can be reversibly oxidized [34]. Given its conservation, we hypothesized that Cys127 in PepMV CP could be involved in stabilizing or regulating the CP-vRNA interaction.

To study how the replacement of Cys127 may affect CP functionality, we used an experimental system based on the PepGFPΔCP mutant, where the ORF encoding the CP was replaced by *GFP* (Fig 1B) [32]. The lack of CP in PepGFPΔCP abolishes cell-to-cell movement and virion formation; the *trans* complementation of PepGFPΔCP by overexpressed CP restores cell-to-cell movement, and can be easily tracked by the formation of fluorescent *foci* [1,32]. Using this experimental system, we previously showed that substitutions of the amino acids that form the clamp found in the CP RNA binding pocket abolished virus cell-to-cell movement; by purifying and analyzing virion preparations, we also showed that these substitutions resulted in no detectable virion formation [1]. Here, we used the same approach to analyze the effect of the Cys127Ser substitution (CP^C127S^). The amino acids cysteine and serine have a similar size and geometry, and their exchange can be considered a very conservative replacement. Two other mutants giving rise to the single replacement Cys127Met (CP^C127M^) or the double replacement Phe126Ile + Cys127Met (CP^F126I+C127M^) were also generated; CPs from members of the family *Potyviridae* have a conserved methionine and a conserved isoleucine in the equivalent positions of PepMV CP Cys127 and Phe126, respectively (S1 Fig). These three CP mutants were tested in *trans*-complementation assays, showing that only the substitution Cys127Ser restored the PepGFPΔCP cell-to-cell movement and produced viral particles, as detected by immunosorbent electron microscopy (ISEM) (Fig 1B). As a control, we expressed the CP wild type (CP^WT^) or CP^C127S^ alone in *Nicotiana benthamiana* leaves; no virus-like particles (VLPs) were observed after ISEM from leaves expressing CP^WT^, while short VLPs were immunocaptured from leaves expressing CP^C127S^ (Fig 1B). As the Cys127Ser replacement produced a functional CP, we generated a full-length PepMV agroinfectious clone bearing *in cis* a mutation giving raise to substitution Cys127Ser in the CP (PepCP^C127S^). PepCP^C127S^ produced a systemic infection in *N*. *benthamiana* plants, and the mutation was stable at least after one plant-to-plant passage (S2 Fig). We then inoculated *N*. *benthamiana* plants with the same amounts of PepCP^C127S^ or PepMV wild type (PepCP^WT^) virion preparations. Ten days post inoculation (dpi), we harvested the four youngest leaves of each plant for relative quantification of viral accumulation. PepCP^C127S^ accumulated almost 60% less than PepCP^WT^ as measured by RT-qPCR (Fig 1C), indicating that the Cys127Ser replacement, even if it was not critical for virus viability, negatively affected viral fitness. Cysteine is the least abundant amino acid in proteins, but multiple roles have been attributed to it based on its unique physiochemical properties and reactivity. Conserved cysteine residues are related to catalytic processes, folding, and metal binding, and also regulation of protein activity through its reversible and interconvertible oxidations that include S-sulfenylation, S-nitrosylation, S-persulfidation, and S-glutathionylation [10]. To determine if Cys127 in PepMV CP was oxidized *in vivo*, we performed thiol alkylation reactions under non-reducing conditions with methyl-polyethylene glycol-maleimide (MM(PEG)_24_) [35] on total protein extracts from *N*. *benthamiana* leaves expressing CP^WT^, CP^C127S^ or PepCP^WT^. In this assay, reduced cysteines are alkylated with MM(PEG)_24_, increasing protein mass by 1.24 kDa per each single conjugation; pre-oxidized (ox) and pre-reduced (red) protein extracts were included as controls. We did not undertake the characterization of cysteine oxidation products directly by mass spectrometry as the products are reversible and unstable. Of note here is that PepMV CP has a single cysteine residue. After immunoblot analysis, a doublet was observed for CP^WT^ (Fig 1D), with the upper band likely corresponding to the alkylated CP, and the lower band, representing approximately one third of the total CP, corresponding to the oxidized CP. No doublet could be observed for CP^C127S^ (Fig 1D). This indicated that the CP^WT^ is oxidized *in vivo*, suggesting that the redox status of the CP may be important during infection. The CP^WT^ expressed from the virus was not completely oxidized or reduced in the controls, suggesting that the CP arranged in virions may not be oxidized or alkylated by MM(PEG)_24_ (S3 Fig).

**Fig 2:**
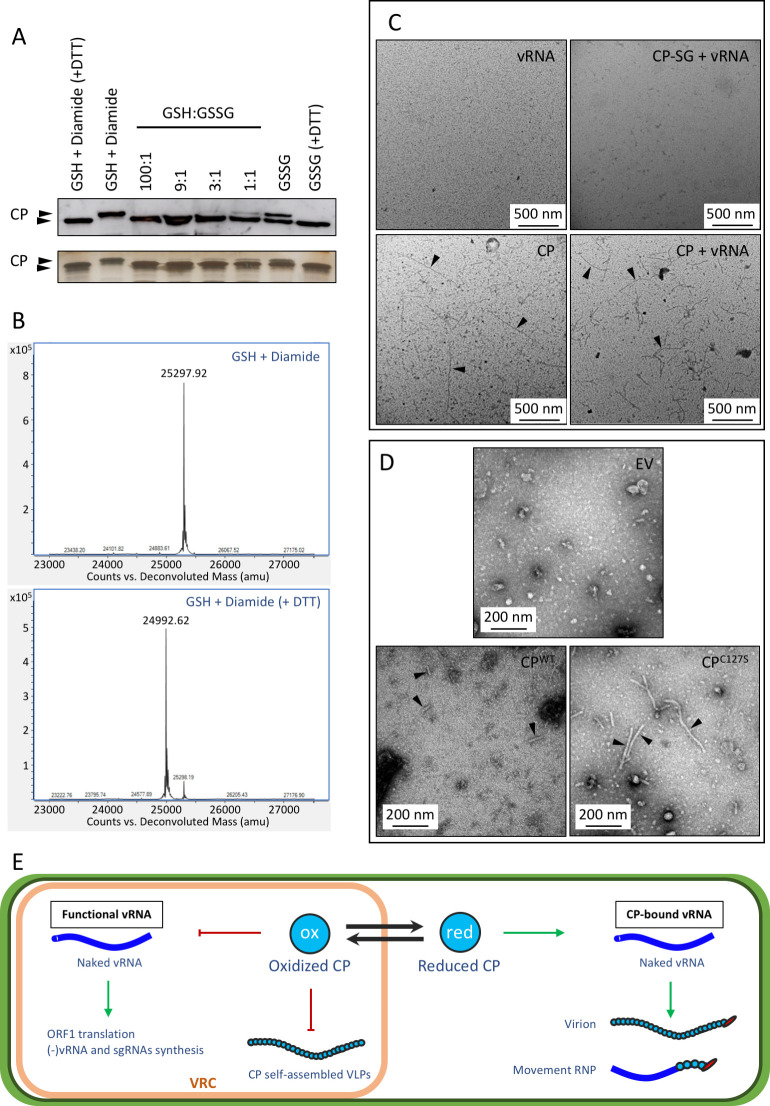
PepMV CP glutathionylation blocks virion formation and CP self-assembly. **(A)** Western blot and silver stained SDS-PAGE to detect the PepMV CP in different *in vitro* glutathionylation reactions. *Escherichia coli*-purified PepMV CP was incubated with reduced glutathione (GSH) + Diamide, oxidized glutathione (GSSG) or different GSH:GSSG ratios; GSH:GSSG ratios 100:1 and 9:1 are similar to those found in cytoplasm and mitochondria, respectively, and 3:1 and 1:1 may be found in the endoplasmic reticulum. An aliquot of the GSH + Diamide and the GSSG reactions were incubated with dithiothreitol (DTT) as the negative control. DTT is a reducing agent that triggers de-glutathionylation of proteins. S-glutathionylation increases protein mass. A total or partial mass shift was observed for the CP incubated with GSH + Diamide or GSSG, respectively, suggesting CP glutathionylation. At a 9:1 ratio of GSH:GSSG, a slight upper band of CP appears in the silver stained gel and the double band is clearly observed at 3:1 and 1:1 ratios. **(B)** Spectra obtained by high-performance liquid chromatography coupled to electrospray ionization and quadrupole time-of-flight mass spectrometry (HPLC-ESI-QTOF-MS) to precisely determine the protein mass of the incubation of the CP with GSH + Diamide and its negative control (+ DTT). S-glutathionylation increases protein mass by 305 Da. The spectra show that CP incubated with GSH + Diamide is 305.3 Da heavier than its negative control, indicating that CP is spontaneously glutathionylated *in vitro* under glutathione redox states similar to those of the endoplasmic reticulum. **(C)** Micrographs of immunosorbent electron microscopy (ISEM) to capture virus-like particles (VLPs) from *in vitro* assembly reactions. Purified RNA from disassembled PepMV virions (vRNA) was incubated alone, with non-glutathionylated recombinant coat protein (CP) or with glutathionylated recombinant CP (CP-SG). As a control, a reaction with only the non-glutathionylated CP was included. VLPs were only immunocaptured from the CP and CP + vRNA reactions and some of them are indicated with an arrowhead. No VLPs were detected by ISEM in the CP-SG + vRNA reaction, indicating that CP glutathionylation blocks virion formation and CP self-assembly. **(D)** ISEM micrographs of crude sap from *N*. *benthamiana* leaves over-expressing CP^WT^ or CP^C127S^ from the pJL-TRBO vector (EV, empty vector). VLPs were immunocaptured from leaves expressing CP^WT^ (indicated with arrowheads) or CP^C127S^ (indicated with arrowheads). CP^C127S^ VLPs were longer than 200 nm while CP^WT^ VLPs were very short and dispersed, indicating the increased ability of the CP^C127S^ mutant to self-assemble *in planta*. A scale bar is drawn in each micrograph, and represents 500 nm (C) or 200 nm (D). **(E)** Proposed model for the regulatory role of CP oxidation during PepMV infection. The reduced coat protein is able to bind viral RNA (vRNA) to form the virion or the movement ribonucleoprotein (RNP). In the virus replication complex (VRC), the CP may be glutathionylated (CP-SG), switching off the CP affinity for the vRNA or the CP oligomerization. The vRNA may thus be available to be used as a template for translation or for the synthesis of the minus-strand and subgenomic RNAs ((-)vRNA and sgRNA, respectively).

Recently, we have described that PepMV infection contributes to hydrogen peroxide and superoxide accumulation in tomato leaves [17]. This accumulation may contribute to oxidation of the CP thiol to a thiyl radical, sulfenic acid, or nitrosothiol, which quickly react with a glutathione molecule (γ-L-glutamyl-L-cysteinyl-glycine; GSH) to form a protein-glutathione disulfide [10,36]. S-glutathionylation is a key post-translational modification in response to oxidants such as reactive oxygen species (ROS), as a redox sensor. S-glutathionylation can occur spontaneously or be enzymatically mediated, and may prevent the over-oxidation of cysteine residues during oxidative stress events, or regulate protein function [37]. Its importance under a range of physiological conditions, including abiotic or biotic stresses, is emerging [9], and can be monitored with reasonable ease. We therefore performed *in vitro* CP glutathionylation assays. *Escherichia coli*-purified CP was incubated under different glutathionylating conditions (GSH+diamide; GSSG), and an aliquot of each reaction was treated with DTT to produce the negative controls. We also prepared CP incubations using different GSH:GSSG ratios to mimic different glutathione redox states from different subcellular compartments, 100:1 and 9:1, which are similar to those found in the cytoplasm and mitochondria, respectively, and 3:1 and 1:1, similar to those found in the endoplasmic reticulum (ER) [36]. After Western blot immunoanalysis and SDS-PAGE gel silver staining, a CP mass shift was observed for the GSH+diamide incubation as compared to its DTT-incubated control (Fig 2A). In the case of the GSSG incubation, a double CP band was observed. A partial CP mass shift was also observed for 9:1, 3:1, and 1:1 GSH:GSSG ratios, with a clear predominance of the lower band (Fig 2A). Further analysis using high-performance liquid chromatography coupled to electrospray ionization and quadrupole time-of-flight mass spectrometry (HPLC-ESI-QTOF-MS) revealed a mass increase of 305.3 Da of the GSH+diamide-incubated CP as compared with the GSH+diamide-incubated CP treated with DTT (Fig 2B). Each glutathione adduct increases protein mass in 305 Da. This result indicates that the CP residue Cys127 is spontaneously glutathionylated under *in vitro* glutathionylating conditions, and partially glutathionylated under glutathione redox states similar to those measured *in vivo* for the ER [38].

The glutathione moiety is large, and therefore, the Cys127 glutathionylation must impose a significant steric hindrance to vRNA binding and encapsidation. To analyze the effect of CP glutathionylation in PepMV particle formation, purified CP was used in *in vitro* VLP assembly assays. The same amounts of non-glutathionylated CP (CP) or glutathionylated CP (CP-SG), produced as before, were incubated with purified vRNA from disassembled PepMV virions. Incubation of vRNA or CP alone were included as controls. Half of each VLP assembly reaction was used to check for the presence of equivalent CP amounts (S4 Fig), and the other half was used for ISEM. No VLPs were observed either in the CP-SG+vRNA incubation or the negative control (vRNA alone), while VLPs were immunocaptured in the CP+vRNA and CP alone incubations (Fig 2C). Extra-long VLPs were observed in the CP incubation, while VLPs from the CP+vRNA incubation seemed to have a shorter and more constant length than VLPs from the CP reaction (Fig 2C). It is worth mentioning that the length of the encapsidated RNA determines the VLP length [22,39]. It is likely that the VLPs with a particle-length similar to that of PepMV virions were vRNA-filled VLPs, while the extra-long VLPs observed in the CP alone incubation were RNA-free VLPs. These results suggest that S-glutathionylation of the CP blocks *in vitro* vRNA encapsidation and CP self-assembly, and are consistent with the observed ability of the non-oxidizable mutant CP^C127S^ to self-assemble *in planta*. Hence, it can be hypothesized that Cys127 S-glutahionylation could modulate CP-vRNA binding, resulting, for instance, in the regulation of viral particle formation.

It is assumed that potexvirus CPs require RNA with an origin of assembly to produce VLPs *in planta* [22]. We observed that glutathionylation blocks *in vitro* CP self-assembly (Fig 2C), and that mutant CP^C127S^ is a non-glutathionylatable although functional CP, which is able to form VLPs (Fig 1B and 1C). These two pieces of evidence strongly suggest that *in vivo* oxidation modulates CP oligomerization. We decided to overexpress the CP^WT^ and the mutant CP^C127S^ *in planta*, using a particularly efficient vector to produce VLPs. We thus overexpressed CP^WT^ or CP^C127S^ in *N*. *benthamiana* leaves with the pJL-TRBO vector [40]; both CPs reached similar expression levels (S5 Fig). Crude sap from agroinfiltrated leaves was used for ISEM. VLPs longer than 200 nm were easily observed from leaves expressing CP^C127S^, while very short and dispersed VLPs were immunocaptured from leaves expressing CP^WT^ (Fig 2D). This is consistent with the fact that CP^WT^ is partially oxidized *in vivo* (Fig 1D), and reduced CP^WT^ may form VLPs. Also, the mutant CP^C127S^ cannot be oxidized and showed an increased ability to form VLPs. This finding has a significant biotechnological interest, since plant VLPs are a promising tool for bio- and nanotechnological applications [41,42]. Our findings can give raise to the first *in planta* platform for efficient VLP production based on a potexvirus CP. Further analyses of CP^C127S^ VLPs may contribute towards the determination of their differences with the PepMV virion.

Taking our results together, we propose a model to explain the biological meaning of CP oxidation during PepMV infection (Fig 2E). A reduced CP is able to bind and encapsidate vRNA to form the movement ribonucleoprotein (RNP) or the virus particle, while an oxidized CP does not have affinity for vRNA, as oxidation affects a critical residue in its RNA binding pocket. The non-encapsidated and naked vRNA is available to be used as a template for ORF1 translation or the synthesis of the minus-strand and subgenomic vRNAs. PepMV builds its replication complex from ER membranes [43]. The ER lumen has an oxidative environment that favors the oxidation of cysteine thiol groups, and we have observed (i) partial *in vivo* CP oxidation (Fig 1D) and (ii) partial spontaneous CP glutathionylation under glutathione redox state conditions similar to those found in the ER (Fig 2A). A plausible hypothesis is that the PepMV CP, within the replication complex, is oxidized to avoid vRNA CP sequestration, to maintain virus replication active. The CP redox status may be spatially and temporally fine-tuned according to the different viral infection stages and scenarios involving enzymes catalyzing CP oxidation or reduction to achieve efficient infection. In this regard, we have identified tomato Tau class glutathione S-transferase 38 (SlGSTU38) as a PepMV proviral factor that interacts and co-localizes with CP within the replication complex [17]; the possibility exists that SlGSTU38 mediates CP S-glutathionylation, thus contributing to the regulation of CP functions.

## Materials and methods

### Plant and virus material

*Nicotiana benthamiana* D. plants grew in a growth chamber set at 25°C with a 16 h/8 h light/dark cycle. The pepino mosaic virus (PepMV) isolate used was Sp13 (GenBank AF484251.1) [44], and the agroinfective clone pBPepXL6 [32] derived from it as the initiator of PepMV infections.

### Structural analysis of the PepMV CP

All the RefSeq sequences of the CPs of members from the four filamentous flexuous families were downloaded from the NCBI database. CPs sequences were aligned using MUSCLE and the consensus sequence logos were built using the WebLogo 3. The atomic model of the PepMV particle was obtained from the protein data bank (accession 5FN1), and the structure of the loose CP subunit was modeled using the AlphaFold2 algorithm from the Colab platform [45]. The pKa and other parameters of the PepMV CP Cys127 residue were calculated using Propka version 2.0 [46] from an on-line platform (https://www.ddl.unimi.it/vegaol/propka.htm). The tridimensional representation of the PepMV CP in the virion context was imaged using PyMol version 2.5.3 (Schrödinger).

### Preparation of DNA constructs

The full genome of PepMV isolate SP13 was amplified with primers 5’ catttcatttggagagggaaaacaaaataaataaataaatatacaaa 3’ and 5’ atgccatgccgaccc(t)_50_ 3’ using pBPepXL6 plasmid [32] as the template and inserted into the pJL89 binary vector using NEBuilder HiFi DNA Assembly Master Mix (New England BioLabs Inc., Ipswich, Massachusetts, United States) following the manufacturers’ instructions, resulting in the pJL89-PepMV vector. Plasmids pDONR/Zeo-CP [17] and pJL89-PepMV were used as the template for site-directed mutagenesis to generate plasmids pDONR/Zeo-CP^C127S^, pDONR/Zeo-CP^C127M^, pDONR/Zeo-CP^F126I+C127M^ and plasmid pJL89-PepCP^C127S^, respectively. First, the PCR products were amplified using the CloneAmp DNA polymerase (Takara Inc., Kusatsu, Japan) with the primers 5’gcaatttt**C**catgtactttgccaaagttgtttgga3’ / 5’tacatg**G**aaaattgcctgggggttatattga3’ to introduce the replacement Cys127Ser, primers 5’ cccccaggcaattt**ATG**atgtactttgccaaag3’ / 5’ ctttggcaaagtacat**CAT**aaattgcctggggg3’ to introduce the replacement Cys127Met, and primers 5’ cccccaggcaa**A**tt**ATG**atgtactttgccaaag3’ / 5’ ctttggcaaagtacat**CAT**aa**T**ttgcctggggg3’ to introduce the replacement Phe126Ile+Cys127Met (mutated nucleotides are indicated in bold and uppercase). PCR products were treated with DpnI, cleaned using NucleoSpin Gel and PCR Clean-up (Macherey-Nagel, Düren, Germany), and circularized with NEBuilder HiFi DNA Assembly Master Mix (New England BioLabs Inc.) following the manufacturers’ instructions. The CP mutants inserts were transferred from the pDONR/Zeo plasmid to the pGWB2 destination vector [47] using Gateway LR Clonase II (Invitrogen, Waltham, Massachusetts, United States). To generate plasmids pJL-TRBO-CP and pJL-TRBO-CP^C127S^, the CP and CP^C127S^ coding sequences (CDSs) were PCR-amplified using primers 5’ttcgtgttcttgtcattaattaacatgcctgacacaac3’ / 5’gttgcaggaccgcggccgccctagttaaagttcagggg3’, and the pJL-TRBO empty vector (EV) [40] was treated with PacI and AvrII. PCR and digestion products were separated by agarose gel electrophoresis and the fragment of interests were excised and cleaned using NucleoSpin Gel and PCR Clean-up (Macherey-Nagel). PCR products were inserted into the pJL-TRBO vector using NEBuilder HiFi DNA Assembly Master Mix (New England BioLabs Inc.) following the manufacturer’s instructions.

### Plant transient transformation

C58C1 *Agrobacterium tumefaciens* cells were transformed with pGWB2- and pJL-TURBO-based vectors. *A*. *tumefaciens* clones were incubated overnight at 28°C in liquid Luria-Bertani (LB) broth complemented with kanamycin and rifampicin until an OD_600_ of 1. Then, the cells were pelleted and resuspended in the same volume of agroinfiltration buffer (10 mM MES pH 5.5, 10 mM MgCl_2_, and 100 mM acetosyringone) and incubated at 25°C with constant stirring for 3 h. Bacterial suspensions were diluted to a final OD_600_ of 0.2 and mixed as required for each experiment. Final suspensions were infiltrated with a needleless syringe on the abaxial side of *N*. *benthamiana* leaves.

### *Trans*-complementation assay

The *trans*-complementation assay was carried out as described by Agirrezabala et al. (2015). Briefly, *N*. *benthamiana* leaves were agroinfiltrated on one half with pBPepGFPΔCP [32] + pGWB2-CP, and on the other half with pBPepGFPΔCP + pGWB2-CP^C127S^, pGWB2-CP^C127M^, or pGWB2-CP^F126I+C127M^. Five days post-agroinfiltration (dpa), the plants were observed under UV light (365 nm) using a handheld lamp (Blak Ray B100-AP lamp, UV products, Upland, California, United States), and images were captured with a Canon EOS 400D camera (Tokyo, Japan).

### Immunosorbent electron microscopy (ISEM)

For immunosorbent electron microscopy (ISEM), a portion of leaf from the *trans*-complementation assay, or agroinfiltrated with pBP19 (expressing the RNA-silencing suppressor protein P19 from tomato bushy stunt virus) + pJL-TRBO-EV, pJL-TRBO-CP or pJL-TRBO-CP^C127S^, was ground with a pestle and a mortar in 2 mL per g of 0.1 M Tris-citric acid pH 8. The homogenate was centrifuged as 3.000 x *g* for 5 min and the supernatant was recovered. *In vitro* assembly reactions (see below) were directly used for ISEM. ISEM was performed as described by Shukla & Gough (1979). Copper electron microscopy grids with carbon film and 400 mesh (Electron Microscopy Sciences, Hatfield, Pennsylvania, United States) were glow-discharged in a Leica EM ACE200 (Leica, Wetzlar, Germany). We used 0.1 mg/mL Protein A (Thermo Fisher Scientific, Waltham, Massachusetts, United States) and a PepMV CP polyclonal antibody raised in rabbit (AC diagnostics Inc., Fayetteville, Arkansas, United States) diluted 1:5 and 1:25 for the first and second incubation, respectively. Grids were contrasted with 2% aqueous uranyl acetate and observed in JEOL 1011 electron microscope (JEOL, Tokyo, Japan) or a Philips Tecnai 12 electron microscope (Amsterdam, Nederland) at an accelerating voltage of 100 kV.

### PepMV and PepCP^C127S^ purification

*N*. *benthamiana* plants were agroinfiltrated with pJL89-PepMV or pJL89-PepCP^C127S^. Systemically-infected symptomatic leaves were harvested at 12 dpa for virus purification. Virus purification was performed as described by Agirrezabala et al. (2015). Virion preparations were resuspended in 0.1 M Tris-citric acid pH 8 and stored at 4°C. Virus suspensions at a OD of 260 nm were measured using a NanoDrop one (Thermo Fisher Scientific) and virus concentration was calculated with ε0.1% = 2.9 as the extinction coefficient [48]. To prepare purified RNA from disassembled PepMV virions, a PepMV preparation was incubated with pronase from *Streptomyces griseus* (Fluka, Buchs, Switzerland) followed by phenol-chloroform purification and RNA ethanol precipitation.

### PepMV RNA accumulation quantification

Dilutions of PepMV or PepCP^C127S^ suspensions at a concentration of 0.1 mg/mL were used to mechanically inoculate 5 *N*. *benthamiana* plants at 5-leaf stage. The first 2 true leaves of each plant were sprinkled with carborundum powder (-400 mesh) (Sigma-Aldrich, St. Louis, Missouri, United States) and inoculated with 10 μL of virus dilution each using a small pestle. Subsequently, the leaves were rinsed with water to remove the carborundum. At 10 days post inoculation (dpi), the 4 youngest leaves of each plant were harvested and ground in a mortar with liquid N_2_. Four mL per g of TNA buffer (0.1 M Tris-HCl pH 8.0, 10 mM EDTA, 2% SDS) were added to the ground leaves and homogenized after the mixture was thawed. Aliquots of 350 μL of the homogenate were used for total RNA extraction with TRI reagent (Molecular Research Center Inc., Cincinnati, Ohio, United States), followed by phenol-chloroform purification and RNA ethanol precipitation. RNA preparations were treated with DNaseI (Sigma-Aldrich), quantified using a NanoDrop One (Thermo Fisher Scientific) and normalized to 10 μL. The RNA quality was analyzed by agarose gel electrophoresis, and 2 RNA preparations of PepCP^WT^ infected plants had to be discarded because of RNA degradation. The relative quantification of PepMV and PepCP^C127S^ was performed individually for each RNA preparation with the one-step NZYSpeedy RT-qPCR Green, ROX plus kit (NZYTech, Lisbon, Portugal) following the manufacturer’s indications, using primers 5’ccccaagtggactgcgttac3’ and 5’gcagcattgtcgtcatcagt3’. The 25S ribosomal RNA (25S) was used as housekeeping RNA and amplified with primers 5’agaactggcgatgcgggatg3’ and 5’gttgattcggcaggtgagttgt3’. The data were analyzed with a one-way ANOVA using the StatGraphics software (StatGraphics Technologies, Inc., The Plains, Virginia, United States). The F-ratio (*F*_x,y_) and p-value (*P*) obtained are indicated in the figure legend.

### PepMV coat protein overexpression in *Escherichia coli*

The *Escherichia coli* production and purification of the CP was carried out by Abyntek Biopharma S.L. (Zamudio, Spain). The CP CDS was optimized for *E*. *coli* and synthesized, and then cloned into the pET-30a(+)vector without any tag fusion. The *E*. *coli* strain BL21 Star (DE3) was transformed with the recombinant plasmid. A clone stored in glycerol was inoculated into Terrific Broth (TB) medium with the appropriate antibiotic and cultured at 37°C. Once the OD_600_ reached 1.2, isopropyl β-d-1-thiogalactopyranoside (IPTG) was added to the cell culture to induce the expression of the recombinant protein and incubated at 15°C for 16 hours. The cells were pelleted by centrifugation, resuspended with lysis buffer, and subjected to sonication. The supernatant after centrifugation was saved for future purification. The target protein was purified through a two-step process using Q sepharose and Superdex 75 columns. The final buffer for protein dilution was 50 mM Tris-HCI, 150 mM NaCl, pH 8.0. The preparation was sterilized with a 0.22 μm filter before being stored in aliquots, and the concentration (1.03 mg/mL) was determined using the Bradford protein assay with BSA as the standard.

### CP redox assay

The CP redox assay was conducted as described by Pant et al. (2021). Disks from *N*. *benthamiana* leaves agroinfiltrated with pGWB2-CP or pGWB2-CP^C127S^ at 4 dpa were used as the starting plant material. Briefly, to prepare the oxidized and reduced controls, plant material was ground in a mortar with liquid nitrogen, homogenized in 4.5 mL / g of lysis buffer (50 mM Tris-HCl pH 7.5, 150 mM NaCl, 10 mM MgCl2, 10% glycerol, 0.1% NP40, 1 mM EDTA, and 1 mM PMSF). The homogenate was centrifuged at maximum speed for 15 min and the supernatant transferred into a new tube. CuCl_2_ to a final concentration of 30 μM, or DTT to a final concentration of 40 mM, were added to a protein extract aliquot, respectively, and incubated at 37°C for 30 min in the case of the oxidized control or 75 min in the case of the reduced control. Then, 10 volumes of trichloroacetic acid (TCA) were added to each control and more plant material was ground in liquid nitrogen and homogenized in 50 mL / g of 10% (v/v) trichloroacetic acid (TCA). After 30 min of incubation in ice, the proteins were pelleted by centrifugation at 16,000 *x g* at 4°C for 10 min. The pellets were washed with 90% and 100% acetone, centrifuged, and air dried. Pellets were resuspended in resuspension buffer (50 mM Tris-HCl pH 7.5, 4 M urea, 2.5% glycerol, 2% SDS, 0.005% bromophenol blue) with methyl-maleimide polyethylene glycol (MM(PEG)_24_) to a final concentration of 1 mM. Tubes were incubated at 65°C for 5 min, and then at 24°C for 1 h. Twenty-five μL of each sample were loaded into an SDS-PAGE gel for Western blot analysis.

### *In vitro* glutathionylation reactions

Five μg of *E*.*coli-*produced CP were incubated at 30°C for 30 min in 50 mM Tris-HCl pH 7.5 with 0.5 mM Diamide plus 0.1 mM GSH, or 10 mM GSSG in 50 μL reactions. After the incubation, each reaction was separated into 2 aliquots of 25 μL, and 2.75 μL of 100 mM DTT were added to one replicate, and 2.75 μL of H_2_O_2_ to the other. The reactions were then incubated at 37°C for 15 min. The GSH and GSSG concentrations in the incubations with different GSH:GSSG ratios were 4.95 mM:0.05 mM (100:1), 4.5 mM:0.5 mM (9:1), 3.75 mM:1.25 mM (3:1), 2.5 mM:2.5 mM (1:1), and 0 mM:5 mM (GSSG). *In vitro* glutathionylation reactions were analyzed by silver stained SDS-PAGE gel, Western blot, or high-performance liquid chromatography coupled to electrospray ionization and quadrupole time-of-flight mass spectrometry (HPLC-ESI-QTOF-MS).

### *In vitro* assembly reaction

A CP + GSH + Diamide glutathionylation reaction (see paragraph above) was scaled up to 400 μL. In parallel, the same amount of protein was prepared without GSH and diamide. After incubation at 30°C for 30 min, both protein preparations were buffer-exchanged into 10 mM Tris-HCl pH 8.5, 30 mM NaCl and concentrated to a final volume of 20 μL using a 10 kDa molecular weight cutoff Ultra-0.5 Centrifugal Filter Unit (Millipore, Burlington, Massachusetts, United States). Ten μL of non-glutahionylated CP (CP) were incubated alone or with 240 ng of purified viral RNA, and 10 μL of glutahionylated CP (CP-SG) were incubated with 240 ng of purified viral RNA at room temperature for 4 h. Five μL of each incubation were analyzed by SDS-PAGE and ISEM.

### Protein electrophoresis and Western blot

Five μL of *in vitro* glutathionylation or assembly reactions were mixed with 2x non-reducing Laemmli loading buffer (300 mM Tris-HCl pH 6.8, 50% Glycerol, 10% SDS; 0.05% bromophenol blue). Total protein extractions of leaves expressing pJL-TRBO-EV, -CP or -CP^C127S^ were performed as described by Méndez-López et al. (2023), and were also mixed with 2x non-reducing Laemmli loading buffer. Samples were loaded in 12% SDS-PAGE gels. The gels were stained with home-made Coomassie blue solution or with Pierce silver stain kit (Thermo Fisher Scientific), or transferred to Amersham Protran 0.45 NC nitrocellulose membranes (Cytiva, Marlborough, Massachusetts, United States) using a Trans-Blot Turbo Transfer System (Bio-Rad, Hercules, California, United States). Blots were probed with an anti-PepMV CP polyclonal antibody raised in rabbit (AC diagnostics Inc.) as the primary antibody, and an anti-rabbit immunoglobulin G (IgG) conjugated to horseradish peroxidase (Promega, Madison, Wisconsin, United States) as the secondary antibody. The immunoreaction was developed using SuperSignal West Femto Chemiluminescent Substrate (Thermo Fisher Scientific) and imaged with an Amersham Imager 680 (GE Healthcare, Chicago, Illinois, United States). For membrane stripping, the membrane was incubated in stripping buffer (62.5 mM Tris-HCl pH 6.8, 2% SDS, 0.1 mM β-mercaptoethanol) for 20 minutes at 50°C, and re-probed with a mouse-produced monoclonal anti-Actin antibody (Sigma-Aldrich).

### High-performance liquid chromatography coupled to electrospray ionization and quadrupole time-of-flight mass spectrometry (HPLC-ESI-QTOF-MS)

The molecular weight of the CP was analyzed using an 1290 Infinity II HPLC (Agilent Technologies, Santa Clara, California, United States) coupled to an iFunnel Q-TOF 6550 system (Agilent Technologies) with a Discovery BIO Wide Pore C5 (2.1x10cm, 5 μm) HPLC column (Supelco Inc., Bellefont, Pennsylvania, United States). Five μL of the glutathionylation reaction were injected with an acetonitrile (ACN) gradient. The predominant peak was analyzed by MS and the mass was obtained after a deconvolution process of the mass over charge (m/z) measurements.

## Supporting information

S1 Table Excel spreadsheet showing the estimates of the pKa, desolvation and interaction with other CP residues of the Cys127 in the virion context or in the loose CP using PROPKA. (XLSX)

S1 Fig Consensus sequence logos of the CP RNA binding pocket domains from the different flexible filamentous virus families. The consensus sequence logos were built using the online tool *WebLogo 3* [49]. The reference sequences (RefSeq) were obtained from the NCBI database, and the number of RefSeq aligned per family is indicated. The conserved invariant amino acids arginine (R) and aspartic acid (D) are highlighted within yellow boxes, and the position of the sulfur-contain amino acid (cysteine or C, and methionine or M) is highlighted in a red box.
(TIFF)

S2 Fig Stability of the single point mutation of clone PepCP^C127S^ after one plant-to-plant passage. The upper part of the panel shows the genomic organization of PepMV. RdRp: RNA dependent RNA polymerase gene; TGBs: Triple gene block protein-encoding genes; CP: coat protein gene. The lower part of the panel shows the alignment of the CP region including the Cys127 codon after genotyping a PepMV-infected plant after one passage (#9), and the 8 PepCP^C127S^-infected plants after one passage (#1–8). The Cys127 codon is framed with a red rectangle. The Cys127 replacement was stable in all the individual passages.
(TIFF)

S3 Fig Redox assay of the PepMV CP expressed from the virus. Western blot of a protein extract from *N*. *benthamiana* leaves expressing PepCP^WT^. Protein extract aliquots were oxidized with CuCl_2_ (lanes 1 and 2), reduced with dithiothreitol (lanes 3 and 4), or not pre-treated with any of them (lanes 5–6). Protein extracts loaded in lanes 2, 4 and 6 were incubated with methyl-polyethylene glycol-maleimide (MM(PEG)_24_) as described by Pant et al. (2021). Reduced cysteines are alkylated with MM(PEG)_24_ and one conjugation increases the protein mass by 1.24 kDa. A double band was observed in lanes 2 and 4, indicating partial CP oxidation, reduction or alkylation in the controls. The membrane was stripped and re-probed with an anti-Actin antibody as the loading control.
(TIFF)

S4 Fig Loading controls for the *in vitro* assembly reactions. Coomassie-stained SDS-PAGE gel showing that similar amounts of PepMV CP were added to each *in vitro* assembly reaction.
(TIF)

S5 Fig Loading controls for the pJL-TRBO-CPs expression experiment. Coomassie-stained SDS-PAGE gel of protein extractions from *Nicotiana benthamiana* leaves expressing the CP wild type (CP) or the CP mutant CP^C127S^. EV: pJL-TRBO empty vector; M: Marker. CP and CP^C127S^ are expressed at similar levels.
(TIF)
