# Supplementary figures and images for: A regulatory role for the redox status of the pepino mosaic virus coat protein
Source: PLoS Pathog. 2023 Oct 18;19(10):e1011732. doi: 10.1371/journal.ppat.1011732 (PMC10615272; doi:10.1371/journal.ppat.1011732)

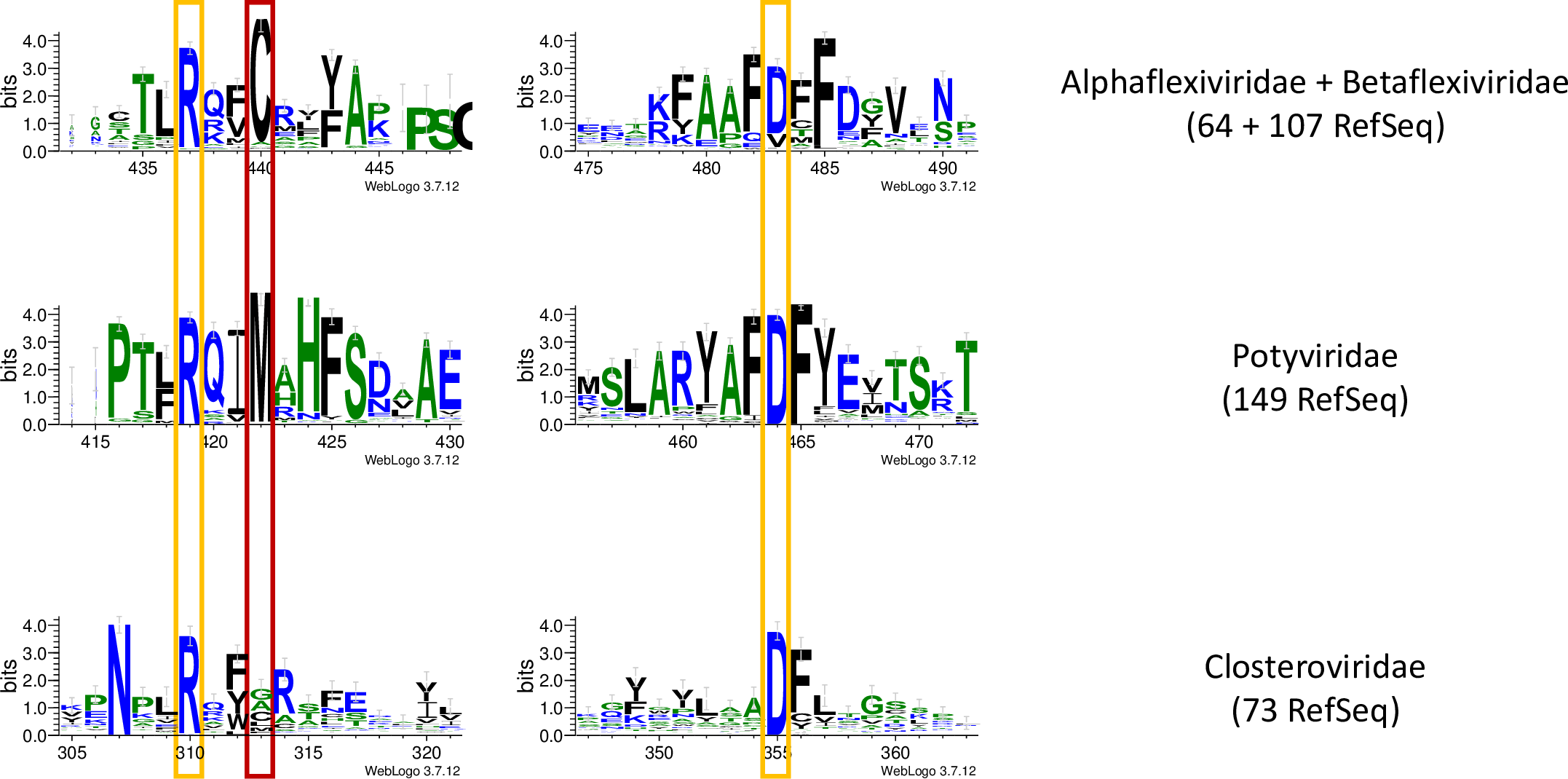

Supplement: S1 Fig — The consensus sequence logos were built using the online tool WebLogo 3 [49]. The reference sequences (RefSeq) were obtained from the NCBI database, and the number of RefSeq aligned per family is indicated. The conserved invariant amino acids arginine (R) and aspartic acid (D) are highlighted within yellow boxes, and the position of the sulfur-contain amino acid (cysteine or C, and methionine or M) is highlighted in a red box. (TIFF) [file ppat.1011732.s002.tiff]

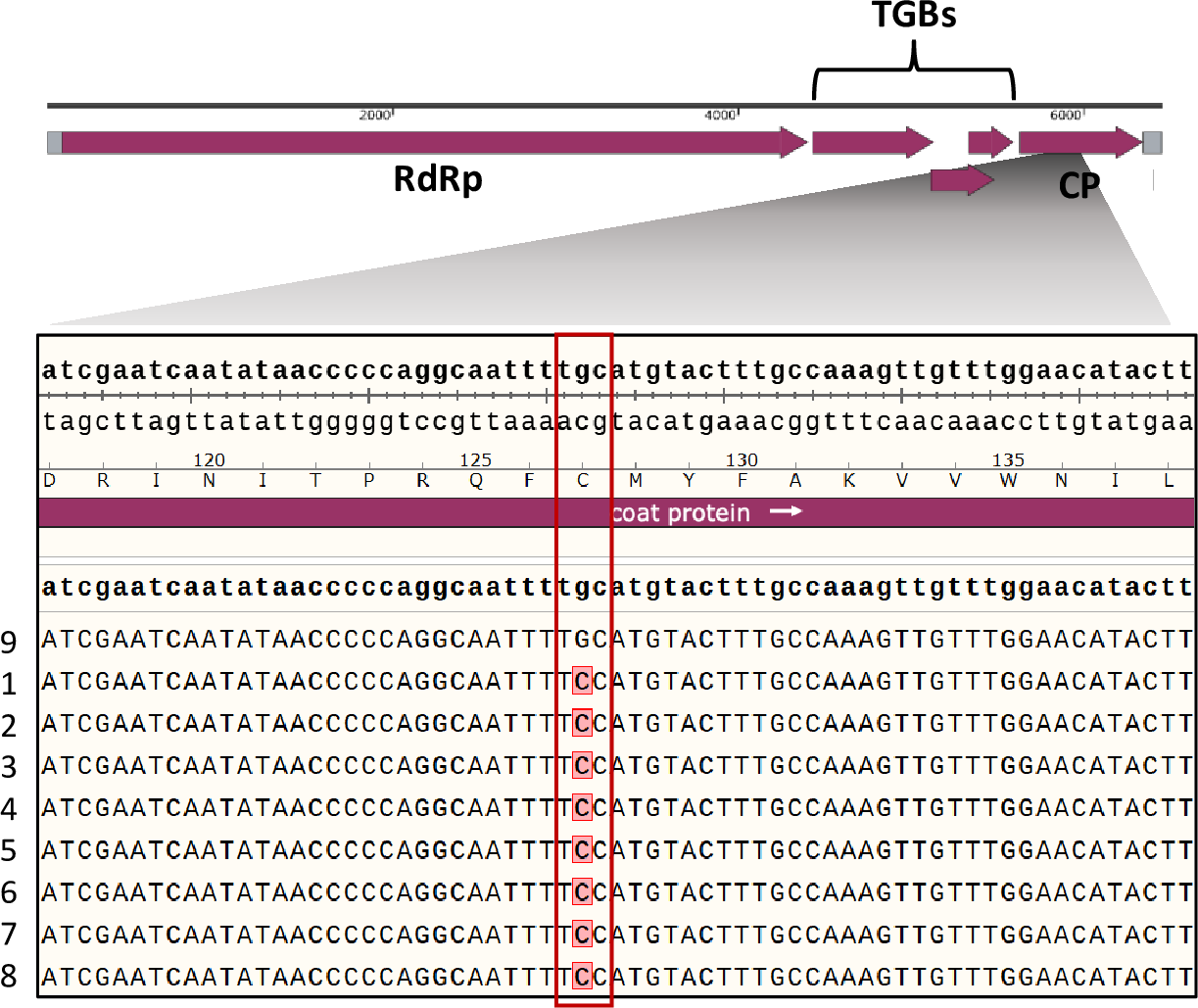

Supplement: S2 Fig — The upper part of the panel shows the genomic organization of PepMV. RdRp: RNA dependent RNA polymerase gene; TGBs: Triple gene block protein-encoding genes; CP: coat protein gene. The lower part of the panel shows the alignment of the CP region including the Cys127 codon after genotyping a PepMV-infected plant after one passage (#9), and the 8 PepCPC127S-infected plants after one passage (#1–8). The Cys127 codon is framed with a red rectangle. The Cys127 replacement was stable in all the individual passages. (TIFF) [file ppat.1011732.s003.tiff]

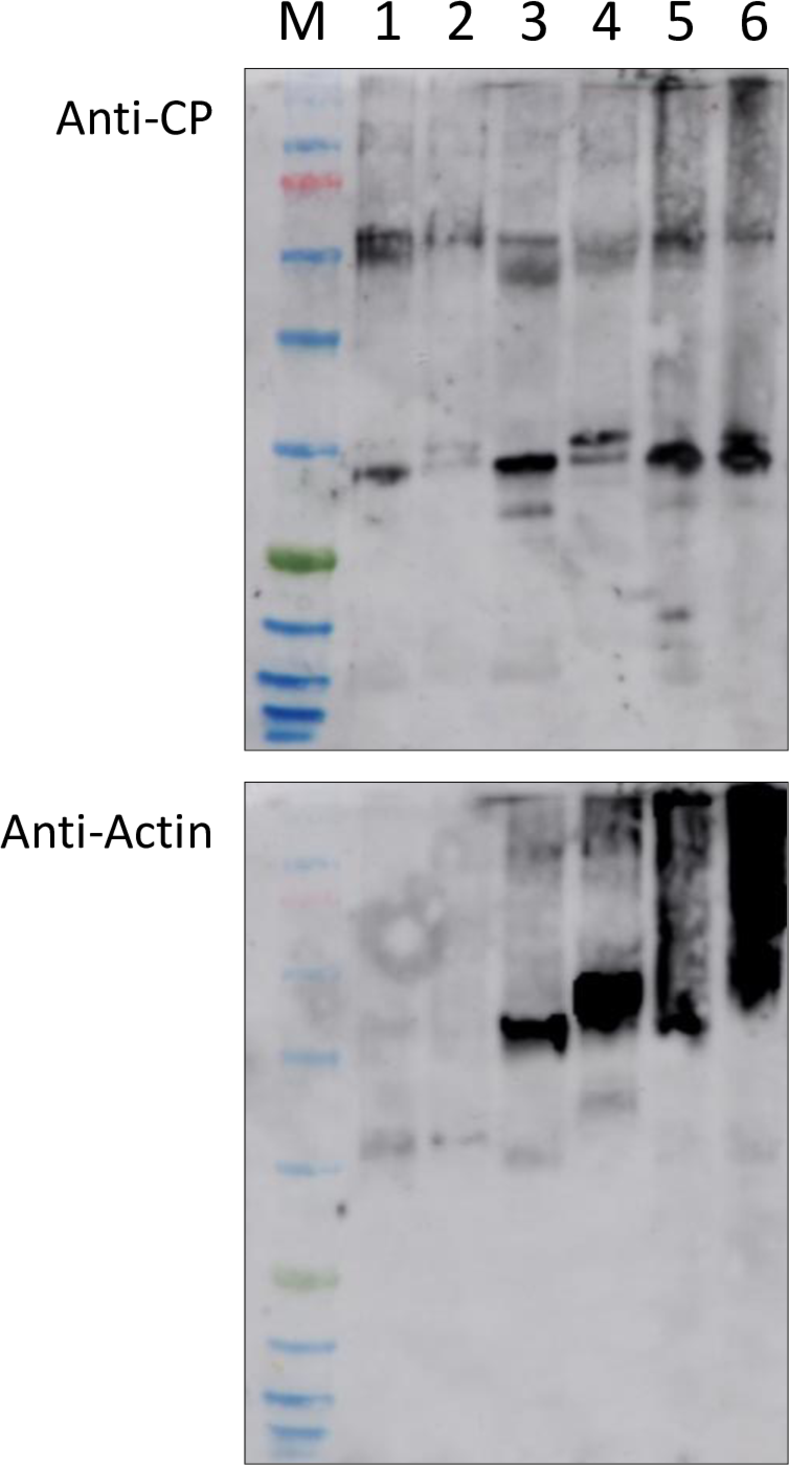

Supplement: S3 Fig — Western blot of a protein extract from N. benthamiana leaves expressing PepCPWT. Protein extract aliquots were oxidized with CuCl2 (lanes 1 and 2), reduced with dithiothreitol (lanes 3 and 4), or not pre-treated with any of them (lanes 5–6). Protein extracts loaded in lanes 2, 4 and 6 were incubated with methyl-polyethylene glycol-maleimide (MM(PEG)24) as described by Pant et al. (2021). Reduced cysteines are alkylated with MM(PEG)24 and one conjugation increases the protein mass by 1.24 kDa. A double band was observed in lanes 2 and 4, indicating partial CP oxidation, reduction or alkylation in the controls. The membrane was stripped and re-probed with an anti-Actin antibody as the loading control. (TIFF) [file ppat.1011732.s004.tiff]

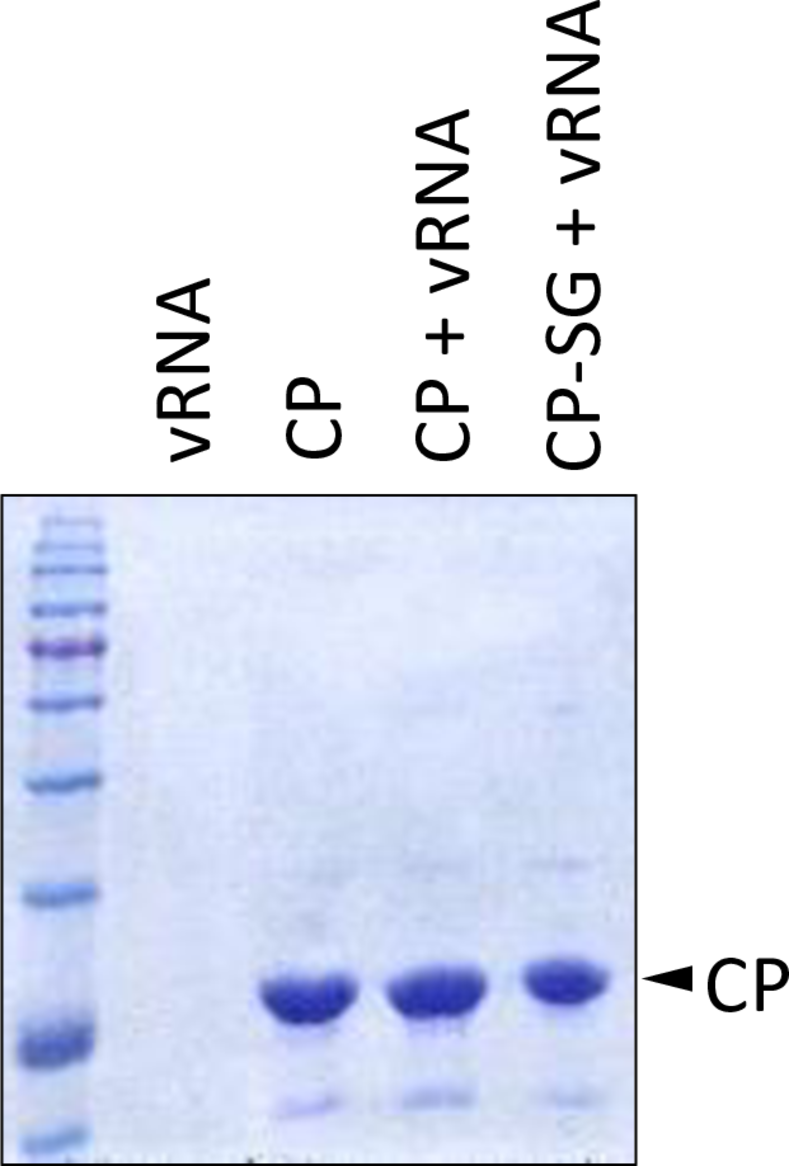

Supplement: S4 Fig — Coomassie-stained SDS-PAGE gel showing that similar amounts of PepMV CP were added to each in vitro assembly reaction. (TIF) [file ppat.1011732.s005.tif]

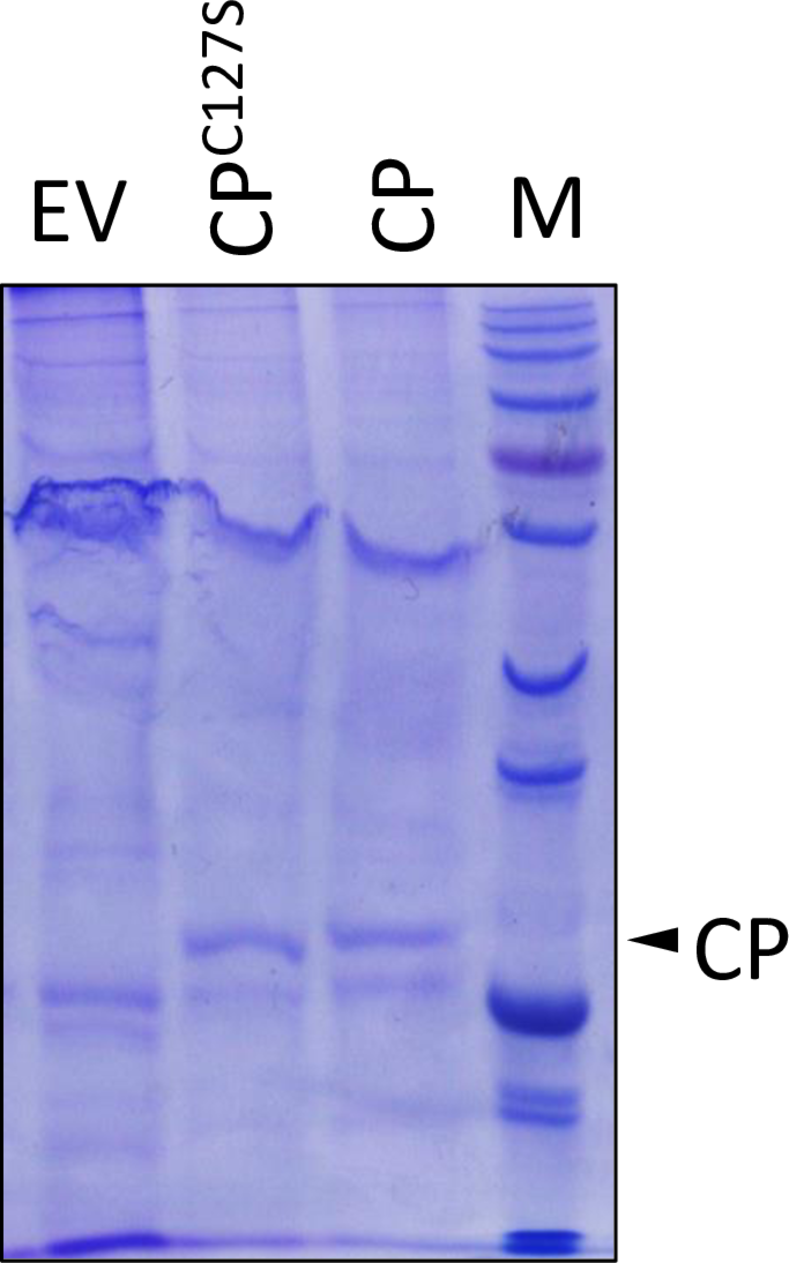

Supplement: S5 Fig — Coomassie-stained SDS-PAGE gel of protein extractions from Nicotiana benthamiana leaves expressing the CP wild type (CP) or the CP mutant CPC127S. EV: pJL-TRBO empty vector; M: Marker. CP and CPC127S are expressed at similar levels. (TIF) [file ppat.1011732.s006.tif]
